# Bcl-2/Bcl-xL inhibitor ABT-263 overcomes hypoxia-driven radioresistence and improves radiotherapy

**DOI:** 10.1038/s41419-021-03971-7

**Published:** 2021-07-13

**Authors:** Violetta Ritter, Franziska Krautter, Diana Klein, Verena Jendrossek, Justine Rudner

**Affiliations:** Institute for Cell Biology (Cancer Research), University Hospital Essen, University of Duisburg-Essen, Essen, Germany

**Keywords:** Cancer microenvironment, Radiotherapy, Cell biology

## Abstract

Hypoxia, a characteristic of most human solid tumors, is a major obstacle to successful radiotherapy. While moderate acute hypoxia increases cell survival, chronic cycling hypoxia triggers adaptation processes, leading to the clonal selection of hypoxia-tolerant, apoptosis-resistant cancer cells. Our results demonstrate that exposure to acute and adaptation to chronic cycling hypoxia alters the balance of Bcl-2 family proteins in favor of anti-apoptotic family members, thereby elevating the apoptotic threshold and attenuating the success of radiotherapy. Of note, inhibition of Bcl-2 and Bcl-xL by BH3-mimetic ABT-263 enhanced the sensitivity of HCT116 colon cancer and NCI-H460 lung cancer cells to the cytotoxic action of ionizing radiation. Importantly, we observed this effect not only in normoxia, but also in severe hypoxia to a similar or even higher extent. ABT-263 furthermore enhanced the response of xenograft tumors of control and hypoxia-selected NCI-H460 cells to radiotherapy, thereby confirming the beneficial effect of combined treatment in vivo. Targeting the Bcl-2 rheostat with ABT-263, therefore, is a particularly promising approach to overcome radioresistance of cancer cells exposed to acute or chronic hypoxia with intermittent reoxygenation. Moreover, we found intrinsic as well as ABT-263- and irradiation-induced regulation of Bcl-2 family members to determine therapy sensitivity. In this context, we identified Mcl-1 as a resistance factor that interfered with apoptosis induction by ABT-263, ionizing radiation, and combinatorial treatment. Collectively, our findings provide novel insights into the molecular determinants of hypoxia-mediated resistance to apoptosis and radiotherapy and a rationale for future therapies of hypoxic and hypoxia-selected tumor cell fractions.

## Introduction

Tumor hypoxia is a common feature in human solid tumors. Chronic hypoxia develops when tumor grows around functional vessels that transport chemically bound oxygen. With increasing distance to the vessel, tumor cells become hypoxic due to diffusion-limited oxygen supply [1]. Occlusion of vessels results in hypoxia due to perfusion-limited oxygen supply. Hypoxic tumor cells, in turn, can activate angiogenesis resulting in the formation of new vessels and reoxygenation. Moreover, tumor vasculature is characterized by a chaotic arrangement, thus providing the tumor insufficiently with oxygen. As a result, tumor cells have to deal with constantly changing oxygen levels that require adaptation and lead to the selection of cells able to handle the microenvironmental stress [2–4]. Moreover, exposure to hypoxia activates several oxygen-sensitive signaling cascades leading to metabolic adaptation, increasing cell death threshold [5] and metastatic potential [6], which finally acounts for a worse response to radio-chemotherapy and poor prognosis. In addition, tumor hypoxia is known to cause resistance to therapeutic agents and to radiotherapy that relies on reactive oxygen species (ROS) production and the formation of irreparable DNA damage as a result of peroxidation events [7–9].

Radiotherapy is broadly applied in anti-cancer therapy, but the development of resistance mechanisms counteracting radiation-induced cytotoxicity remains a major problem in the treatment of cancer patients. Previous work described that ionizing radiation (IR) can induce apoptosis, necrosis, mitotic cell death, as well as senescence and other forms of cell death [10, 11]. Among these, radiation-induced apoptosis was intensely studied over the last two decades. Generally, IR activates intrinsic apoptosis pathway that is regulated at the mitochondrial level by members of the B cell leukemia (Bcl)-2 family [12, 13]. Based on their action, Bcl-2 family members are divided in pro- and anti-apoptotic proteins, the former facilitating cytochrome C release from mitochondria and subsequent caspase activation while the latter prevents it [14]. Activation of pro-apoptotic multidomain proteins Bax and Bak is indispensable for cytochrome C release and can be prevented by interacting with anti-apoptotic Bcl-2 members. Activation of Bax and Bak is further regulated by pro-apoptotic Bcl-2 homology domain (BH)3-only proteins either by direct interaction with these pro-apoptotic multidomain proteins or by sequestration of anti-apoptotic Bcl-2 family members [14]. In the end, the tightly controlled balance between pro-apoptotic and anti-apoptotic Bcl-2 proteins, called Bcl-2 rheostat, decides about cytochrome C release from mitochondria and apoptosis induction, thereby participating in therapy response [14, 15].

Analyzing the role of anti-apoptotic Bcl-2 family members in hypoxia-mediated radioresistance, we demonstrate here that exposure of cancer cells to acute and repeated cycles of hypoxia alters the balance of Bcl-2 rheostat in favor of anti-apoptotic family members, thereby elevating the apoptotic threshold, impeding cell death induction and restricting favorable therapeutic outcome. However, hypoxia-mediated radioresistance could be overcome by BH3-mimetic ABT-263 targeting anti-apoptotic Bcl-2 and Bcl-xL. Hypoxia-mediated upregulation of anti-apoptotic Mcl-1 can pose an additional factor decreasing sensitivity to radiation-induced apoptosis. Targeting Mcl-1 further increased apoptosis sensitivity to ABT-263 alone or combined with radiotherapy. ABT-263 did not only countervail hypoxia-mediated radioresistance, but, beyond that, further enhanced the response of xenograft tumors of non-selected and hypoxia-selected NCI-H460 cells to radiotherapy, thereby confirming the beneficial effect of combined treatment in vivo. Targeting the Bcl-2 rheostat with ABT-263 is therefore a promising approach to overcome the radioresistance of tumor cells exposed to acute hypoxia or selected by cycling exposure to hypoxia.

## Results

### Exposure to acute and cycling hypoxia reduces radiation-induced apoptosis and the efficacy of radiotherapy

**Fig. 1:**
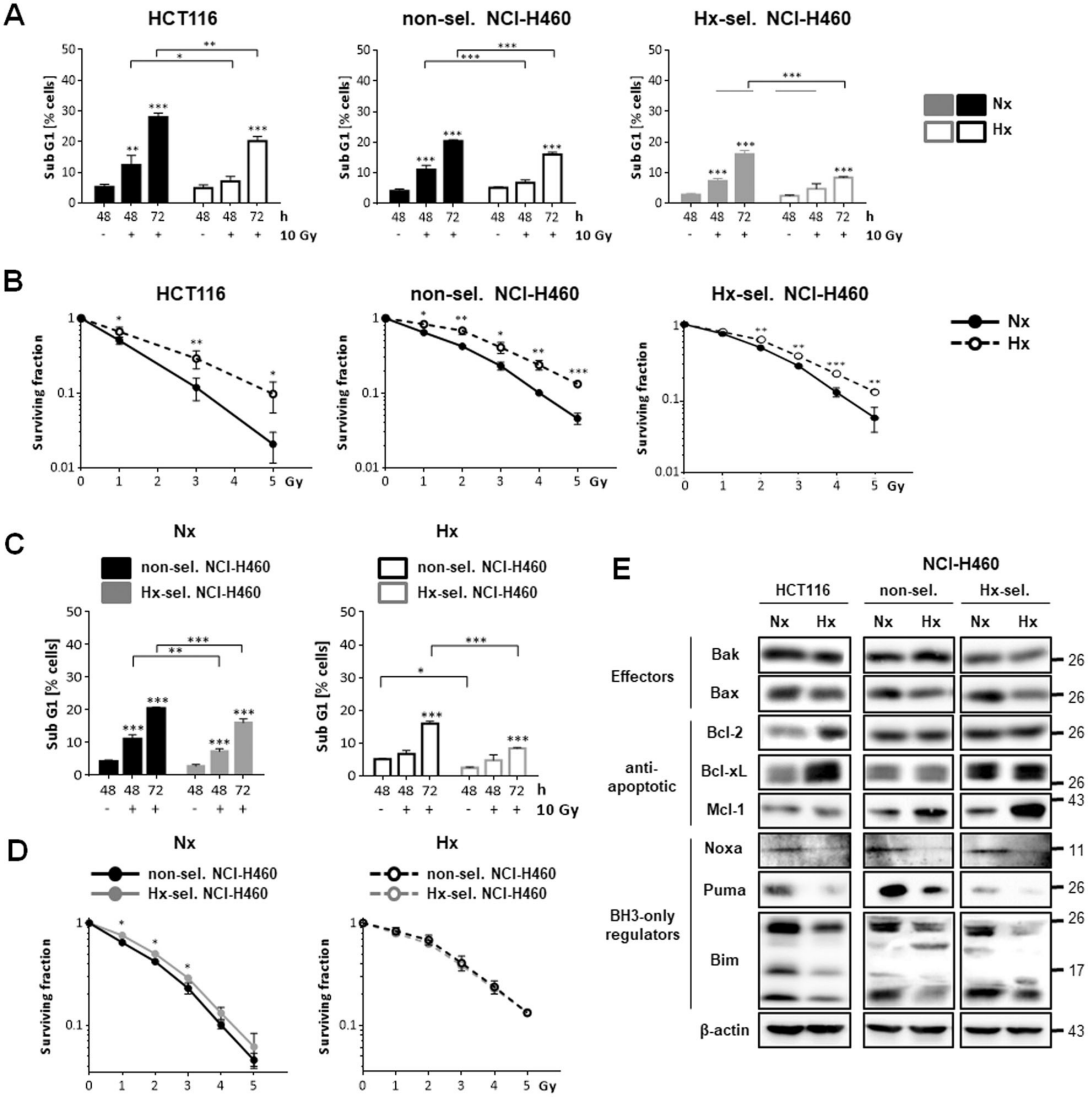
Exposure to acute and adaptation to cycling hypoxia results in reduced radiation-induced apoptosis, enhanced clonogenic survival, and altered balance of pro- and anti-apoptotic Bcl-2 protein family members. Human HCT116 colon carcinoma cells and non-selected and hypoxia-selected NCI-H460 lung adenocarcinoma cells were irradiated under normoxic (Nx, 20% O_2_) or severely hypoxic conditions (Hx, <0.2% O_2_). **A** 48 and 72 h after irradiation with a single dose of 10 Gy, apoptosis induction was assessed by flow cytometric analysis of DNA fragmentation (Sub G1 fraction). **B** Following irradiation with 0–5 Gy in normoxia, cells were incubated for 12 days in normoxia. Irradiation in severe hypoxia was followed by incubation in hypoxia for 48 h before transfer to normoxia for another 10 days. Surviving fractions were calculated as ratio of seeded cells to counted colonies and normalized to respective untreated controls. Data in (**A**) and (**B**) compare the effect in response to irradiation acute hypoxia to that in normoxia. **C** Direct comparison of apoptosis induction of non-selected and hypoxia-selected NCI-H460 cells irradiated under normoxic and hypoxic conditions. **D** Direct comparison of clonogenic survival of non-selected and hypoxia-selected NCI-H460 cells irradiated under normoxic and hypoxic conditions. Data in (**C**) and (**D**) compare the effect of irradiation in non-selected versus hypoxia-selected NCI-H460 cells. **E** Changes of protein levels of pro- and anti-apoptotic Bcl-2 protein family members in HCT116 cells and both NCI-H460 cell lines exposed to normoxia or severe hypoxia for 24 h were determined at the protein level by Western blot analysis. Protein levels of non-selected and hypoxia-selected NCI-H460 cells were analyzed on the same membranes or at similar detection conditions. β-actin was used as loading control. Figures show representative results. Data are shown as mean values of at least three independent experiments ± SD. *P ≤ 0.05; **P ≤ 0.01; ***P ≤ 0.001. **A**, **C** *, **, *** above bars: comparing irradiated to non-irradiated respective controls. **B**, **D** *, **, ***: comparing effects of irradiation at respective doses in normoxia to that in hypoxia. Same data sets were used in (**A**) and (**C**) as well as in (**B**) and (**D**).

At first, we aimed to verify the relationship between tumor hypoxia, apoptosis resistance, and response to radiotherapy described in previous studies [16, 17]. To analyze the effect of acute hypoxia on radioresistance and apoptosis resistance, we irradiated HCT116 colon carcinoma and NCI-H460 lung adenocarcinoma cells in normoxic or severe hypoxic (<0.2% O_2_) conditions. To examine the effect of cycling hypoxia, we employed hypoxia-selected NCI-H460 cells that were established by exposing them to repeated cycles of severe hypoxia and intermittent reoxygenation [18]. Irradiation of HCT116 cells and both NCI-H460 cell lines in normoxia induced apoptosis in a time-dependent manner starting 48 h and clearly increasing 72 h after irradiation. Irradiation in hypoxic conditions resulted in significantly lower apoptosis rates than in normoxic conditions (Fig. 1A). Moreover, clonogenic survival decreased with increasing radiation doses in normoxia and severe hypoxia (Fig. 1B), but irradiation in severe hypoxia resulted in significantly higher surviving fractions (SF) in all three cell lines.

Compared to non-selected NCI-H460 cells, radiation-induced apoptosis was significantly reduced while clonogenic survival was slightly but significantly increased (Fig. 1D) in hypoxia-selected NCI-H460 cells when irradiation was applied in normoxia.

Our data reveals that exposure to acute hypoxia or repetitive cycles of hypoxia/reoxygenation improved cell survival after irradiation and increased radioresistance.

### Exposure to acute and adaptation to cycling hypoxia alters Bcl-2 rheostat

Hypoxia tolerance and resulting clonal evolution of therapy-resistant cancer cells are promoted through alterations in the mitochondrial apoptosis program [19–21]. Thus, we analyzed the expression of Bcl-2 protein family members in non-selected and hypoxia-selected NCI-H460 cells exposed to acute hypoxia for 6 h or in normoxia. A microarray-based analysis revealed that the expression of Bcl-2 family members was changed in NCI-H460 cells exposed to acute hypoxia or to repeated cycles of hypoxia/reoxygenation (Fig. S1). Gene expression profiles differed more prominently under normoxic than under hypoxic conditions, and these of hypoxia-selected cells displayed greater changes than those of non-selected cells.

Next, protein levels of several Bcl-2 family members were investigated by Western blot analysis in HCT116 and NCI-H460 cells exposed to normoxia and severe hypoxia (Fig. 1E). Protein levels of the pro-apoptotic BH3-only regulators Noxa, Puma, and Bim and of apoptosis effector Bax were reduced in all three cell lines exposed to hypoxia. Regulation of anti-apoptotic proteins was different in HCT116 and NCI-H460 cells in response to acute hypoxia. While Bcl-2 and Bcl-xL were strongly upregulated in hypoxic HCT116 cells, Mcl-1 levels did not change. In non-selected and hypoxia-selected NCI-H460 cells exposed to acute hypoxia, Mcl-1 levels were strongly upregulated while Bcl-2 and Bcl-xL levels did not change. Moreover, we detected higher levels of anti-apoptotic Bcl-xL and lower levels of pro-apoptotic Bak in hypoxia-selected than in non-selected NCI-H460 cells.

These results evidence that acute and chronic cycling hypoxia affected the Bcl-2 rheostat.

### ABT-263 improves radiation-induced apoptosis in normoxic, hypoxic, and hypoxia-selected cancer cells

**Fig. 2:**
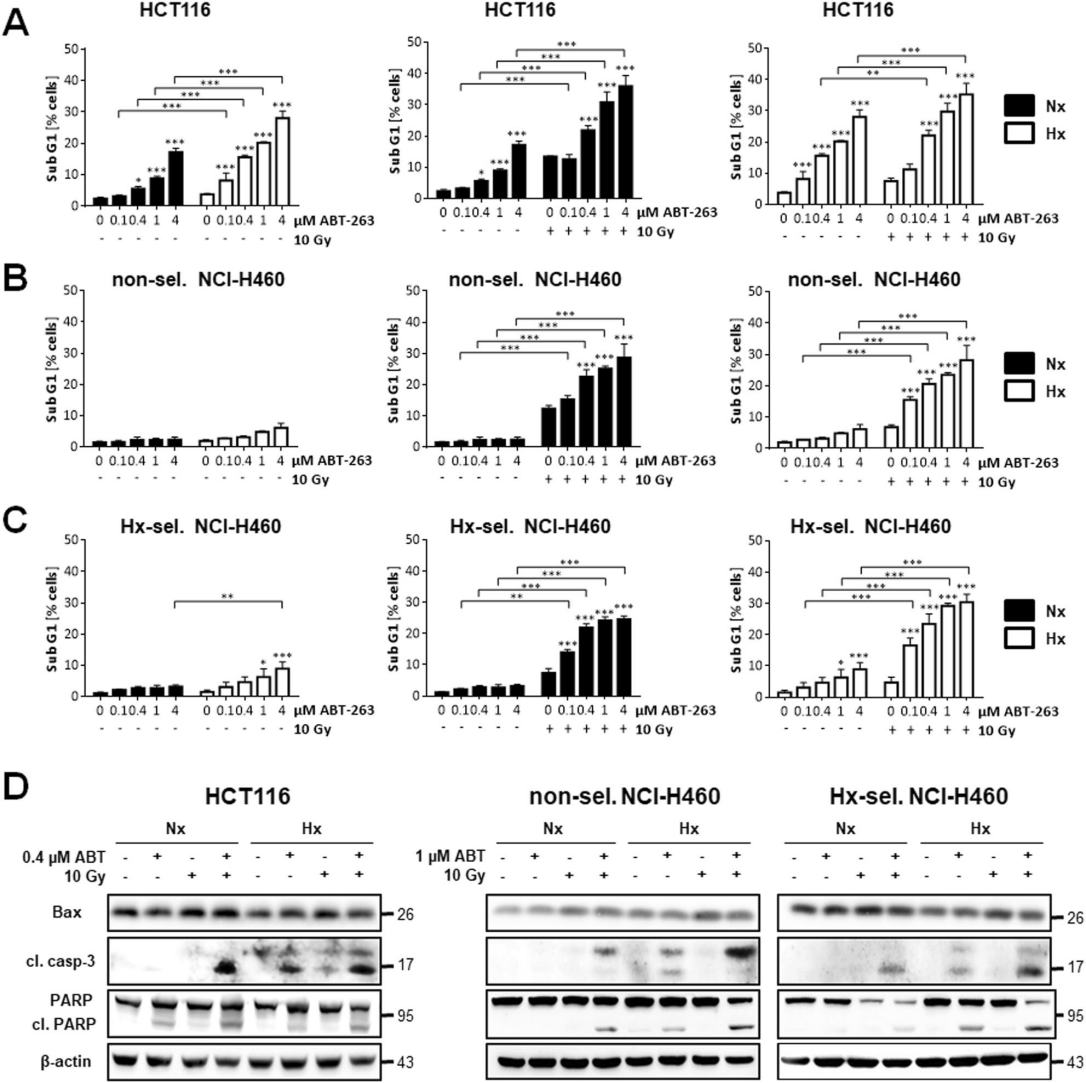
ABT-263 induces apoptosis and enhances radiation-induced apoptosis in HCT116 as well as non-selected and hypoxia-selected NCI-H460 cells. Cells were treated with indicated concentrations of ABT-263, irradiated with 10 Gy (IR), or both in normoxia (Nx, 20% O_2_) or in severe hypoxia (Hx, <0.2% O_2_). Apoptosis levels of (**A**) HCT116, (**B**) non-selected NCI-H460 and (**C**) hypoxia-selected NCI-H460 cells were determined by flow cytometric analysis of DNA fragmentation (Sub G1 fraction) 48 h after respective treatment in normoxic or hypoxic condition. Data are shown as mean of three independent experiments ± SD. *P ≤ 0.05; **P ≤ 0.01; ***P ≤ 0.001. *, **, *** above bars: comparing treatment with ABT-263 to non-treated respective controls (0 µM ABT-263). Same data sets were used in left and middle graphs to show apoptosis in normoxic cells. Similarly, same data sets were used in left and right graphs to show apoptosis in hypoxic cells. **D** Whole-cell lysates were made 48 h after respective treatment and protein levels of cleaved caspase 3, PARP, cleaved PARP, and Bax were assessed by Western blot analysis. β-actin was used as loading control for whole-cell lysates. Figures show representative results.

From our preceding experiments, we hypothesized that the shift of Bcl-2 rheostat towards anti-apoptotic members in acute and cycling hypoxia promotes resistance to apoptosis and increases long-term survival upon irradiation, thereby impairing the effect of radiotherapy. Shifting the balance back to pro-apoptotic Bcl-2 family members by targeting anti-apoptotic Bcl-2/Bcl-xL using ABT-263 should therefore overcome hypoxia-mediated resistance to radiotherapy and improve the therapeutic outcome. Flow cytometric analysis of DNA fragmentation revealed that treatment with ABT-263 for 48 h induced apoptosis in a dose-dependent manner in HCT116 cells (Fig. 2A). ABT-263-induced apoptosis was significantly higher in HCT116 cells treated in severe hypoxia than in normoxia. Additional irradiation clearly augmented apoptotic rates in HCT116 cells. Interestingly, combined therapy-induced apoptosis similarly effective in normoxia and in severe hypoxia. Compared to HCT116 cells, non-selected and hypoxia-selected NCI-H460 cells hardly induced apoptosis following ABT-263 treatment in normoxia or hypoxia (Fig. 2B and C, respectively), but apoptosis was clearly induced by ABT-263 when irradiation was applied. The combined therapy was similarly effective in normoxia and hypoxia in both, non-selected and hypoxia-selected NCI-H460 cells.

Apoptosis induction was further verified by analyzing cleavage of caspase-3 and the caspase-3 substrate PARP (Fig. 2D). For this purpose, HCT116 cells were treated with 0.4 µM ABT-263, while more resistant NCI-H460 cells were treated with 1 µM ABT-263. Alternatively, cells were irradiated with 10 Gy and irradiation was combined with ABT-263 treatment. Conform to apoptosis induction assessed by flow cytometry, the strongest apoptotic cleavage of caspase-3 and its substrate PARP was detectable upon combined treatment with ABT-263 and IR in all three cell lines. In HCT116 cells, a considerable caspase-3 and PARP cleavage was observed when combined treatment was applied in normoxia, but cleavage was not enhanced in hypoxia. In non-selected and hypoxia-selected NCI-H460 cells, combined treatment resulted in moderate caspase-3 and PARP cleavage, but caspase-3 and PARP cleavage were enhanced when treatment was performed in hypoxia.

**Fig. 3:**
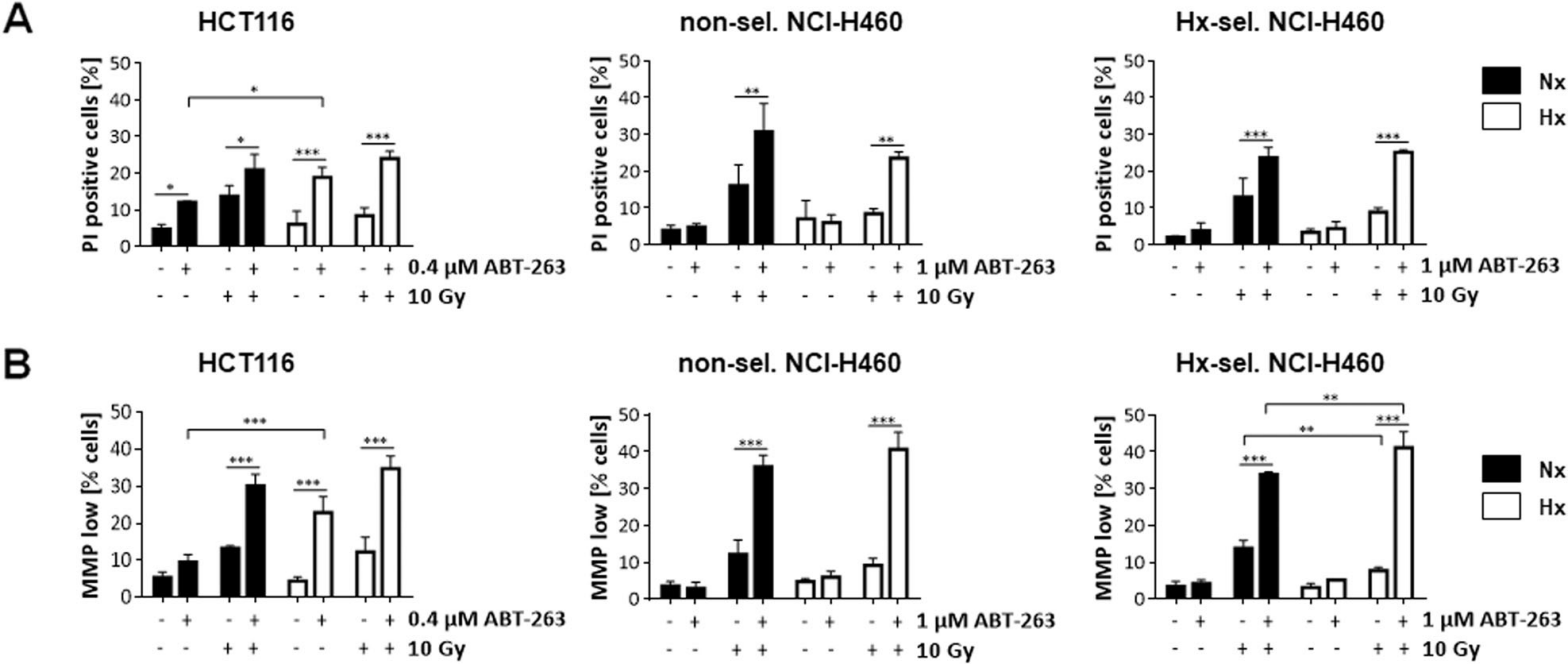
Cell death induction in response to ABT-263 and ionizing radiation. Cells were treated with indicated concentrations of ABT-263, irradiated with 10 Gy (IR), or both in normoxia (Nx, 20 % O_2_) or in severe hypoxia (Hx, <0.2% O_2_). 48 h later, cell death induction was assessed by flow cytometric analysis using (**A**) propidium iodide exclusion staining (PI-positive cells) and (**B**) TMRE to detect dissipation of mitochondrial membrane potential (MMP low). Data are shown as mean of three independent experiments ± SD. *P ≤ 0.05; **P ≤ 0.01; ***P ≤ 0.001.

Next, we examined cell death induction in general employing propidium iodide exclusion assay (Fig. 3A) and measuring the dissipation of mitochondrial membrane potential (MMP) (Fig. 3B) 48 h after treatment with ABT-263 (HCT116: 0.4 µM, NCI-H460: 1 µM), irradiation with 10 Gy, or the respective combined therapy. Combined therapy induced cell death more effectively than either single therapy. Furthermore, combined treatment with ABT-263 and IR was similarly effective in normoxia as in hypoxia. The cell death rates in response to ABT-263-based therapies were comparable to apoptosis rates, indicating that HCT116 and NCI-H460 cells mainly underwent apoptosis.

### Treatment with ABT-263 and IR alters Mcl-1 protein levels

**Fig. 4:**
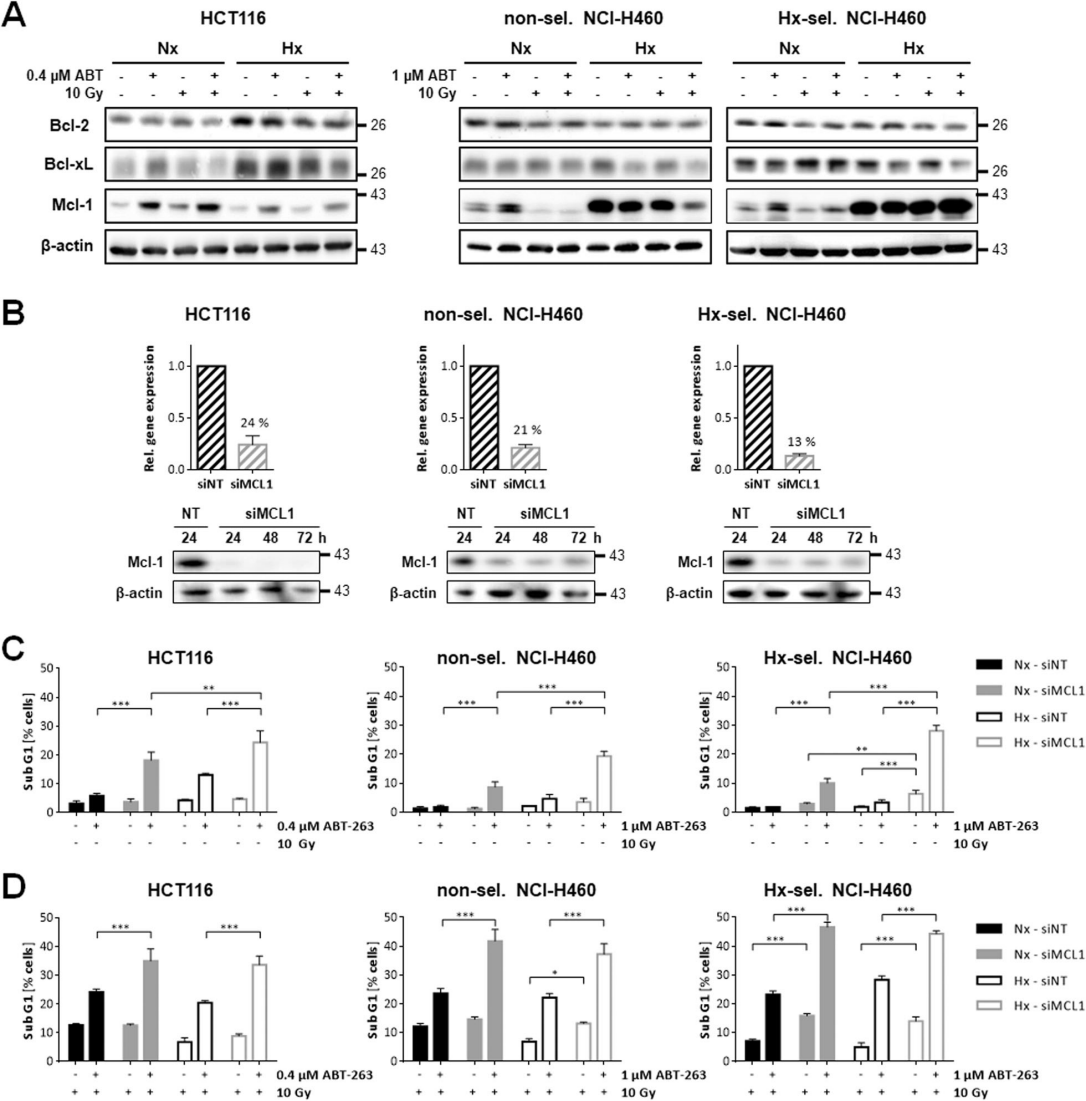
Silencing of *MCL1* increases sensitivity to ABT-263 and combinatory therapy of ABT-263 and ionizing radiation. **A** Changes of anti-apoptotic proteins Bcl-2, Bcl-xL, and Mcl-1 were examined by Western blot 48 h after treatment with ABT-263, irradiation, or combined treatment in HCT116 and non-selected and hypoxia-selected NCI-H460 cells under normoxic (Nx, 20% O_2_) or in severe hypoxic (Hx, <0.2% O_2_) conditions. β-actin was used as loading control. Figures show representative results. **B**–**D** Cells were transfected with *MCL1*-targeting (siMCL1) or non-targeting control siRNA (siNT). **B** Knockdown efficiency was assessed by qRT-PCR and Western blot analysis at indicated time points after transfection. **C** 24 h after transfection, the cells were treated with ABT-263 in normoxia (Nx; 20% O_2_) or severe hypoxia (Hx; 0.2% O_2_) or **D** additionally irradiated (IR) with 10 Gy. Apoptosis levels (Sub G1 fraction) were determined 48 h after respective treatment. Data show mean values of three independent experiments ± SD. *P ≤ 0.05; **P ≤ 0.01; ***P ≤ 0.001.

The therapeutic effect of ABT-263 is based on the neutralization of anti-apoptotic Bcl-2 and Bcl-xL [22], but could be overcome by high levels of Mcl-1, which is not a specific target of ABT-263 [13, 23]. We therefore analyzed protein levels of Bcl-2, Bcl-xL, and Mcl-1 48 h after treatment with ABT-263 (HCT116: 0.4 µM, NCI-H460: 1 µM), IR (10 Gy) or combined therapy (Fig. 4A). While Bcl-2 levels were generally higher in HCT116 exposed to hypoxia, they were reduced particularly in response to the combined therapy. Bcl-2 levels hardly changed in non-selected and hypoxia-selected NCI-H460 cells treated with ABT-263 and IR either in normoxia or hypoxia. Similar to Bcl-2, the combined therapy was particularly effective in lowering hypoxia-induced Bcl-xL level in HCT116 cells. In non-selected and hypoxia-selected NCI-H460 cells, treatment with ABT-263 alone reduced Bcl-xL level. In hypoxia-selected NCI-H460 cells, the combined treatment in hypoxia reduced Bcl-xL even better than treatment with ABT-263 alone.

The regulation of Mcl-1 levels in response to the Bcl-2/Bcl-xL inhibitor and irradiation, however, was quite unexpected. In HCT116 cells, Mcl-1 levels increased after treatment with ABT-263 alone or in combination with radiotherapy, but the increase was less pronounced when treatment was performed in hypoxia than in normoxia. In non-selected and in hypoxia-selected NCI-H460 cells, Mcl-1 levels were also increased after treatment with ABT-263 in normoxia, but ABT-263-induced Mcl-1 accumulation was abrogated when combined therapy was applied. A strong Mcl-1 accumulation was detected in both NCI-H460 cell lines exposed to hypoxia. In non-selected NCI-H460 cells, hypoxia-induced Mcl-1 levels were partially reduced after treatment with ABT-263 or irradiation and effectively in response to combined treatment. In contrast, hypoxia-induced Mcl-1 levels remained high in hypoxia-selected NCI-H460 cells even after the combined treatment.

### Silencing of *MCL1* sensitizes tumor cells to apoptosis induced by ABT-263 alone or in combination with radiotherapy in normoxia and hypoxia

Since ABT-263 is not a specific inhibitor of Mcl-1, elevated Mcl-1 levels can impair ABT-263-mediated cytotoxicity. In the next set of experiments, we therefore aimed to silence Mcl-1 expression in order to increase apoptosis induction in response to ABT-263-based therapy regiment. Successful siRNA-mediated silencing of *MCL1* gene expression was verified in all three cell lines using qRT-PCR and Western blot analysis (Fig. 4B). *MCL1* gene expression was clearly reduced in non-selected and hypoxia-selected NCI-H460 cells 24 h after transfection, while Mcl-1 protein levels were not detectable in HCT116 cells. MCL-1 levels remained low for another 48 h. Thus, 24 h after transfection, cells were treated with ABT-263 alone (Fig. 4C) or with an additional radiotherapy (Fig. 4D). Flow cytometric analysis of DNA fragmentation revealed that MCL1 knockdown did not affect viability in all three cell lines in normoxia, and increased only mildly above background levels in hypoxia-selected NCI-H460 cells exposed to severe hypoxia. Silencing of MCL1 gene expression sensitized all three cell lines to ABT-263-induced apoptosis. The sensitizing effect was significantly better in hypoxia compared to normoxia. Additional irradiation with 10 Gy further increased ABT-263-induced apoptosis to similar rates in normoxia and hypoxia.

### Co-treatment with ABT-263 overcomes radioresistance acquired by acute or cycling hypoxia

**Fig. 5:**
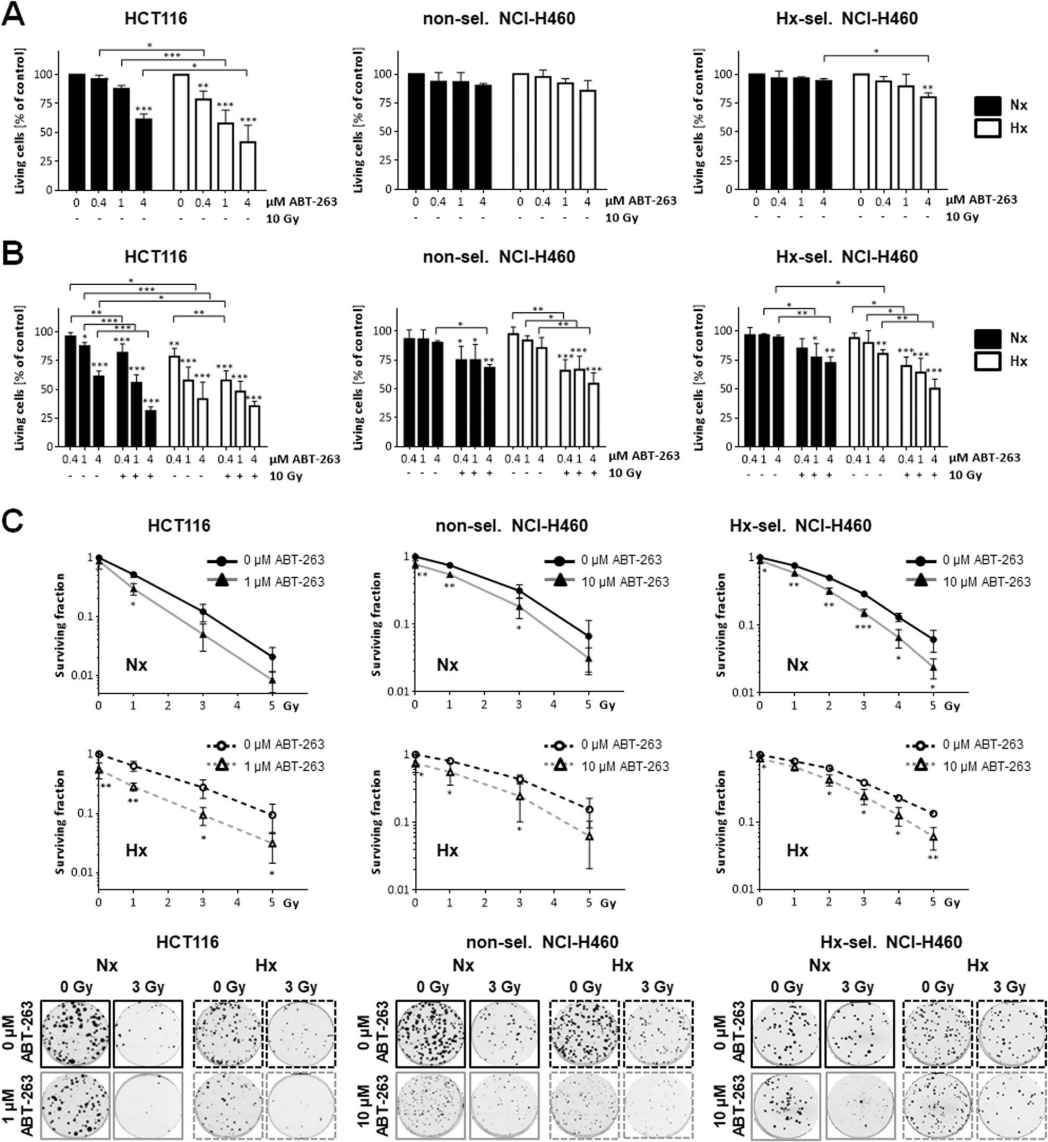
Combined treatment with ABT-263 and ionizing radiation reduces short-term and long-term-term survival in response to acute hypoxia in hypoxia-selected cells. **A**, **B** HCT116 cells and non-selected and hypoxia-selected NCI-H460 cells were treated as indicated with ABT-263, ionizing radiation (IR), or both in normoxia (Nx, 20% O_2_) or in severe hypoxia (Hx, <0.2% O_2_). After 48 h, attached cells were fixed and stained with crystal violet solution. Spectrophotometrical measurement of released dye after cell lysis was used to calculate the amount of viable cells by normalizing the values to untreated controls or irradiation-only treated cells. **A** Short-term survival after treatment with ABT-263 and **B** after additional irradiation. **C** Upper surviving curves: Following treatment with ABT-263 and irradiation under normoxic conditions, the cells were incubated in normoxia for 12 days. Lower surviving curves: Following treatment with ABT-263 and irradiation under hypoxic conditions, the cells were kept in severe hypoxia for 48 h before transfer to and further incubation in normoxia for 10 days. Formed colonies (bottom, representative images) were counted and surviving fractions were calculated. Data show mean values of at least three independent experiments ±SD. *P ≤ 0.05; **P ≤ 0.01; ***P ≤ 0.001. **A**, **B** *, **, *** above bars: comparing treatment with ABT-263 to non-treated respective controls (0 µM ABT-263) at respective conditions. **C** *, **, ***: comparing effects after treatment with ABT-263 to these after treatment with solvent (0 µM ABT-263) at respective conditions.

To further assess the therapeutic potential of ABT-263 alone and in combination with radiotherapy, we analyzed the short-term and long-term survival of the cancer cell lines in response to treatment employing crystal violet assay and a clonogenic assay, respectively. Treatment with 0.4 µM ABT-263 already significantly reduced the short-term viability of HCT116 cells in hypoxia, while higher ABT-263 concentrations (4 µM) were required to significantly reduce cell survival in normoxia (Fig. 5A). Treatment of non-selected and hypoxia-selected NCI-H460 cells with ABT-263 hardly affected short-term survival. Only when treated with the highest ABT-263 concentration (4 µM) in hypoxia, short-term survival of hypoxia-selected cells was significantly reduced.

Additional irradiation further reduced the short-term survival of HCT116 cells treated with ABT-263 in normoxia (Fig. 5B). In hypoxia, an additional cytotoxic effect of irradiation was only observed when a low ABT-263 concentration (0.4 µM) was applied. Irradiation also reduced short-term survival after ABT-263 treatment of non-selected and hypoxia-selected NCI-H460 cells. Here, the toxic effect of combined therapy was more pronounced in hypoxia than in normoxia. Based on ABT-mediated cytotoxicity in short-term assays, we chose to treat HCT116 cells with 1 µM ABT-263 and NCI-H460 cells with 10 µM ABT-263 in long-term survival assays (Fig. 5C) to ensure that enough ABT-263 was still active after prolonged incubation at 37 °C.

While treatment of HCT116 cells with the Bcl-2/Bcl-xL inhibitor did not affect surviving fraction (SF) in normoxia, it significantly reduced SF in hypoxia. We also detected a significantly reduced SF after treatment with ABT-263 in normoxia and hypoxia in non-selected as well as hypoxia-selected NCI-H460 cells. In all three cell lines, we observed lower SF values after the combined therapy than after radiotherapy alone, particularly when a lower irradiation dose was used.

In summary, our results revealed that inhibition of anti-apoptotic Bcl-2/Bcl-xL can improve the cytotoxic response to radiotherapy in vitro.

### Treatment with ABT-263 overcomes radioresistance acquired by cyclic exposure to hypoxia in NCI-H460 xenograft mouse model

**Fig. 6:**
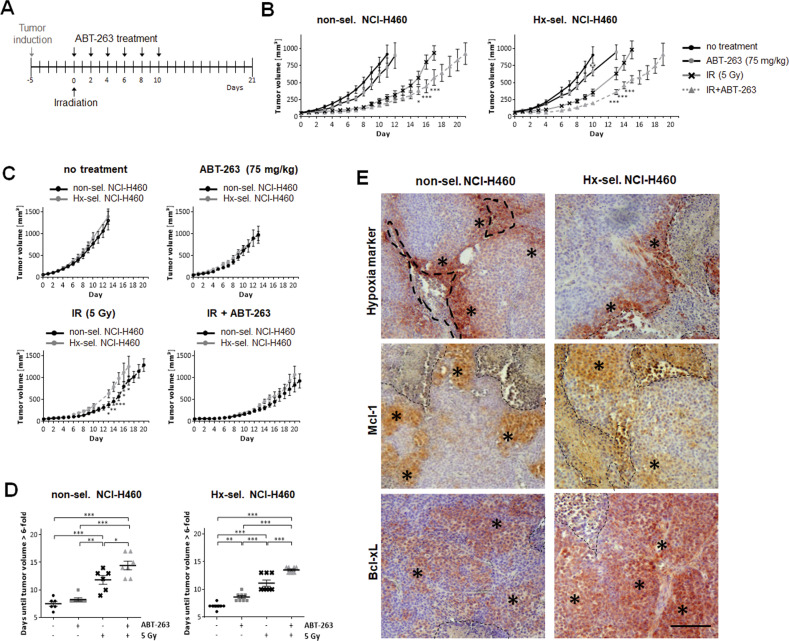
Combined treatment with ABT-263 and ionizing radiation decelerates growth of non-selected and hypoxia-selected NCI-H460 xenograft tumors. **A** Treatment schedule: When the tumor volume reached 50 mm³, xenograft tumors of non-selected and hypoxia-selected NCI-H460 cells were irradiated with a single dose of 5 Gy. ABT-263 (75 mg/kg) was applied via intraperitoneal injection every other day (six injections in total), starting 2 h after tumor irradiation. **B** Growth of NCI-H460-derived tumors comparing different treatment modalities. **C** Comparing growth of tumors derived from non-selected and hypoxia-selected NCI-H460 cells. **D** Analysis of days until tumors reaches sixfold volume. Data shown in (**B**–**D**) presents mean tumor volume of 6–8 animals per group ± SEM. *P ≤ 0.05; **P ≤ 0.01; ***P ≤ 0.001. **B** *, **, ***: comparing effects of combined therapy (IR + ABT-263) with these of radiotherapy alone (IR) at indicated days. **C** *, **, ***: comparing effects on tumors derived from non-selected (non-sel.) with these derived from hypoxia-selected (Hx-sel.) NCI-H460 cells. **E** Immunohistochemical analysis of non-selected and hypoxia-selected NCI-H460 tumors using a hypoxia marker or antibody against Mcl-1 and Bcl-xL (brown staining). Viable and stained tumor cells are emphasized by asterisks. dashed lines mark necrotic areas. Scale bar: 50 µm.

Finally, we intended to examine the combined effect of ABT-263 and radiotherapy in mouse tumor xenografts. In vivo experiments were confined to hypoxia-selected and non-selected NCI-H460 cells. Both NCI-H460-derived tumors grew with similar kinetics (Fig. 6B–D). Immunohistochemical analysis revealed that both NCI-H460-derived tumors developed hypoxic areas that were commonly found around necrotic areas (Fig. 6E). In the vicinity of these necrotic regions, we detected cells with elevated Mcl-1 expression. A stronger Bcl-xL immunoreactivity was detected in hypoxia-selected tumors compared to non-selected counterparts and was not restricted the perinecrotic regions.

Treatment started when tumors reached 50 mm^3^ (day 0) (Fig. 6A). Untreated tumors established from non-selected and hypoxia-selected NCI-H460 cells grew with similar kinetics. Treatment with ABT-263 alone delayed tumor growth only minimally, while irradiation alone resulted in a pronounced growth delay of tumors derived from in both cell variants (Fig. 6B). Still, irradiated hypoxia-selected NCI-H460 tumors grew significantly faster than the control counterparts (Fig. 6C), verifying in vivo the increased radioresistance of cells exposed to cycling hypoxia. Combined therapy delayed tumor growth even more efficiently than either single therapy (Fig. 6B). Direct comparison of non-selected and hypoxia-selected NCI-H460 tumors revealed similar growth rates in response to the combined therapy (Fig. 6C). Furthermore, a significantly prolonged time until tumors reached the 6-fold volume was calculated after combined treatment than after either single treatment (Fig. 6D), supporting the results shown in Fig. 6B.

Thus, the in vivo experiments demonstrate that combined treatment with ABT-263 and IR can overcome radioresistance acquired after cyclic exposure to hypoxia and can delay tumor growth beyond the radioresistance factor.

## Discussion

Over the past decades, radiotherapy strongly focused on technique refinement, in order to enable the precise deposition of an effective dose to the tumor while sparing normal tissue [24, 25]. However, despite valuable technical improvements and the substantial therapeutic benefit achieved in individual tumors, many tumors respond poorly to radiotherapy. One major factor limiting tumor response to IR is hypoxia [6]. DNA damage induced by photon-based radiotherapy is greatly impaired in hypoxic environment. Moreover, cycling hypoxia can increase genomic instability and result in the accumulation of clones with altered metabolism, increased DNA repair and anti-oxidative capacity as well as resistance to cell death and metastatic behavior [6, 17, 18, 26, 27]. In our studies, we intended to mimic hypoxic situations found in vivo by analyzing the cellular response to radiotherapy in HCT116 colon cancer and NCI-H460 lung adenocarcinoma cells exposed acutely to severe hypoxia and in NCI-H460 cells exposed to repeated cycles of hypoxia/reoxygenation, thus allowing accumulation of cells with high tolerance to fluctuating oxygen levels. The hypoxia-selected cells displayed reduced radiosensitivity compared to their non-selected counterparts, but exposure to acute hypoxia further enhanced radioresistance to a similar extent as in non-selected counterparts. Increased radioresistance was also observed in HCT116 colon cancer cells exposed to acute hypoxia in vitro. Furthermore, employing mouse xenograft model, we demonstrated that irradiated hypoxia-selected NCI-H460 tumors grew faster than non-selected control tumors. Taken together, we could reproduce the resistance-promoting effects of acute hypoxia and cycling hypoxia on radiotherapy in in vitro and in vivo models.

Exposure of tumor cells to cycling hypoxia can result in clonal selection of cells with improved survival mechanisms and tolerance to hypoxia/reoxygenation, which often goes together with reduced sensitivity to radiotherapy and other anti-neoplastic therapies [17, 28]. We identified deregulated Bcl-2 rheostat after exposure to acute hypoxia as well as chronic cycling hypoxia resulting in decreased levels of pro-apoptotic and increased levels of anti-apoptotic proteins. Downregulated pro-apoptotic Bcl-2 family members were previously reported for several cancer cell lines exposed to acute hypoxia [20, 29], suggesting that downregulation of pro-apoptotic Bcl-2 family members may be a general response of cancer cells to hypoxia and contribute to hypoxia-mediated apoptosis resistance. On the other hand, upregulation of anti-apoptotic Bcl-2 family members was likewise associated with hypoxia-mediated evasion of apoptosis in a cell type- and context-dependent manner in several studies [19, 30]. Moreover, a deregulated Bcl-2 rheostat is often observed in human tumors, including lung and colon cancer [14, 31–33].

Importantly, our findings on an altered Bcl-2 rheostat correlated with increased resistance to radiation-induced apoptosis in hypoxia and in hypoxia-selected NCI-H460 cells. This implied that interfering with the Bcl-2 rheostat by shifting the balance towards pro-apoptotic proteins might lower the cell death threshold and restore radiosensitivity. We could reproduce the sensitizing effect of ABT-263 to radiotherapy in HCT116 and NCI-H460 cells in vitro and in vivo in murine xenograft model. More importantly, we demonstrated that hypoxia-mediated deregulation of Bcl-2 rheostat is causative for a worse response to radiotherapy and thus represents a promising target to improve the therapeutic outcome.

Along with Bcl-2 and Bcl-xL, anti-apoptotic Bcl-2 family member Mcl-1 regulates response to ABT-263-based therapies [13, 23, 34]. Previous observation described that human cancer cells were sensitized to apoptosis induced by Bcl-2/Bcl-xL-targeting BH3 mimetics via hypoxia-mediated downregulation of Mcl-1 [23]. In contrast, we demonstrated that acute hypoxia increased Mcl-1 levels especially in hypoxia-selected NCI-H460 cells, while Mcl-1 level did not change in HCT116 cells. A sensitization to ABT-263 by downregulation of Mcl-1 was described in many other studies [13, 35–37]. Apparently, the radiosensitizing effect of ABT-263 depends on Mcl-1 expression and/or stability. In response to irradiation, deubiquitinase USP9x can stabilize Mcl-1 stability [13, 35], thereby reducing ABT-263-mediated cytotoxicity. We, however, detected downregulation of Mcl-1 after irradiation in normoxia in both hypoxia-selected and non-selected NCI-H460 cells. In hypoxia, IR-induced downregulation of Mcl-1 was detected only in non-selected NCI-H460 cells. In normoxia as well as in hypoxia, ABT-263-induced cytotoxicity was comparable in irradiated non-selected in hypoxia-selected NCI-H460 cells and did not correlate with Mcl-1 levels.

Finally, ABT-263 can increase the stability of Mcl-1 mRNA and protein as previously described in hepatocellular carcinoma cells [38], suggesting that treatment with ABT-263 alone might activate resistance mechanisms by increasing anti-apoptotic Mcl-1. We also detected higher Mcl-1 protein levels upon treatment with ABT-263 especially in HCT116 cells. However, the higher Mcl-1 protein level could not be associated with increased resistance to ABT-263 in normoxia or in hypoxia.

The orally available Bcl-2/Bcl-xL inhibitor ABT-263 is a drug mimicking BH3-only protein Bad [22]. A safe application with few side effects and promising outcome in lung cancer patients was initially shown in clinical phase 1 and 2 studies [39, 40]. Results from the following clinical trials evaluating the therapeutic benefit of Bcl-2/Bcl-xL-targeting BH3-mimetics indicate that these inhibitors may be particularly useful in combination with conventional chemotherapeutic agents [41–43]. Combinatory treatment with IR was not examined in clinical trials, yet a precise local treatment by IR in combination with a systemic application of the radiosensitizing Bcl-2/Bcl-xL inhibitor seems exceptionally promising. Several preclinical investigations already demonstrated synergistic effects of BH3-mimetics in combination with IR [42, 44]. The effectivity of BH3-mimetica alone or in combination with cytotoxic drugs was also demonstrated in hypoxia in several cancer cells including neuroblastoma, lung and colon carcinoma cells [23, 45]. Combinatory treatment with BH3-mimetics and radiotherapy is therefore a promising approach that should be evaluated in clinical studies, as this therapy concept could be of great benefit for patients with hypoxic tumors refractory to radiotherapy. However, thrombocytopenia is the major side effect limiting the ABT-263 dose in anti-tumor therapies and commonly observed in extended-field radiotherapy [39, 40, 46]. Concurrent chemotherapy and a higher amount of irradiated bone marrow increase risk of thrombocytopenia particularly in extended-field therapy [46]. The mice we used in our experiment tolerated the combined therapy very well. We irradiated the tumor-bearing hind leg only and spared the marrow. Although the new irradiation techniques allow a very precise irradiation of tumors, these known side effects need monitoring after a prolonged radiotherapy, particularly when combined with ABT-263.

In summary, we demonstrated that exposure to acute or cycling hypoxia changed the Bcl-2 rheostat and increased resistance to radiotherapy in NCI-H460 lung cancer and HCT116 colon cancer cells. Targeting the Bcl-2 rheostat by ABT-263 not only improved the cytotoxic effect of radiotherapy on cancer cells in normoxic conditions, but particularly overcomes radioresistance of cancer cells exposed to acute or cycling hypoxia in vitro and in vivo.

## Materials and methods

### Cell lines and treatment

Colon carcinoma cell line HCT116 and the lung adenocarcinoma cell line NCI-H460 were obtained from ATCC (Bethesda, MD, USA). Cells were grown in RPMI 1640 medium supplemented with 10% fetal calf serum (Gibco/Life Technologies, Carlsbad, CA, USA) and kept in a humidified incubator at 37 °C and 5% CO_2_. Treatment and cultivation in hypoxic conditions were performed in a humidified hypoxia workstation (Ruskinn Technology, Bridgend, UK) at 37 °C, <0.2% O_2_, and 5% CO_2_. Prior to any treatment, cells, media, and solutions were equilibrated to hypoxic conditions for 2 h. The hypoxia-selected NCI-H460 sub-cell line was generated as described before [18]. Before freezing several stocks of cells, cell identity was verified using short tandem repeat analysis. After thawing, cells were analyzed for mycoplasma contamination. Morphology was checked weekly using microscopy.

Cells were irradiated with X-ray machine X-RAD 320 (Precision X-Ray, North Branford, CT, USA) operated at 320 kV, 10 mA with a 1.65 mm aluminum filter at room temperature with an effective photon energy of 90 kV at a dose rate of 2.75 Gy/min. For irradiation under hypoxic conditions, plates or dishes containing cells were transferred to airtight pouches. For combined treatment, ABT-263 (Gentaur, Aachen, Germany) was added directly after irradiation.

### Flow cytometry

Cells were harvested and incubated with appropriate staining solutions in the dark for 30 min at 37 °C. Dissipation of the MMP was examined using the MMP-specific dye tetramethylrhodamine ethyl ester (TMRE, Molecular Probes, distributed by Thermo Fisher Scientific, Grand Island, NY, USA), applied at 25 nM in PBS. To quantify apoptotic DNA fragmentation (Sub G1), cells were incubated with PBS containing 0.1% sodium citrate, 0.1% Triton X-100, and 50 µg/ml propidium iodide (Gibco/Life Technologies, Carlsbad, CA, USA). Cell death was quantified after staining the cells with 10 µg/mL propidium iodide in PBS. Flow cytometric measurements were performed with a BD Accuri™ C6 flow cytometer (Becton Dickinson Bioscience, Heidelberg, Germany) and analyzed with respective BD Accuri™ C6 software.

### Colony formation assay

Cells were seeded (100–6400 cells/well) in six-well plates. After treatment with ABT-263 and/or irradiation, cells were incubated for 12 days in normoxia. To access clonogenic survival in hypoxia, cells adjusted to the hypoxic condition 2 h before treatment and were left in hypoxia for another 48 h before transfer to normoxic conditions for further 10 days. Then, cells were fixed with 3.7% formaldehyde and 70% ethanol and stained with 0.05% Coomassie brilliant blue. Colonies of more than 50 cells were counted using GelCount™ colony counter and respective software (Oxford Optronix, Oxfordshire, Great Britain). SFs were calculated by normalizing the ratio of seeded cells to counted colonies after treatment to that of untreated control sample.

### Analysis of proteins and mRNA

For protein detection, cell were lysed and western blot analysis was performed as described before [13]. To detect mRNA, qPCR was performed as described before [47] using following primers: Mcl-1: MCL1for (TCTCATTTCTTTGGGTGCCTTT), MCL1rev (GATATGCCAAACCAGCTCCTAC); House keeping gene β2-microglobulin: B2Mfor (TGCTGTCTCCATGTTTGATGTATCT), B2Mrev (TCTCTGCTCCCCACCTCTAAGT).

### siRNA-mediated gene silencing

Cells were transfected with *MCL1*-directed siRNA (ON-TARGET SMARTpool) or non-targeting siRNA (NON-TARGETING pool) purchased from Dharmacon (Chicago, IL, USA) using Trans-IT siQuest transfection reagent (Mirus, Madison, WI, USA) according to the manufacturer’s protocol. The cells were further treated or analyzed after overnight transfection.

### Mouse xenograft tumor generation and in vivo treatment

Mouse experiments were carried out in strict accordance with the recommendations of the Guide for the Care and Use of Laboratory Animals of the German Government and they were approved by the Committee on the Ethics of Animal Experiments of the responsible authorities (registration number AZ 84-02.04.2014.A481). Xenograft tumors were generated by subcutaneous injection of 5 × 10^5^ cells onto the hind leg of NMRI-nu/nu mice (total volume 50 µl) as previously described [48, 49]. Tumor volume was determined at indicated time points using a sliding caliper. When tumor volume reached 50 mm³, mice were randomly allocated to treatment groups of 6–8 animals each. For radiation therapy, mice were anesthetized (2% isoflurane) and tumors were exposed to a single dose of 5 Gy in 5 mm tissue depth (∼1.5 Gy/min, 300 kV, filter: 0.5 mm Cu, 10 mA, focus distance: 60 cm) using a collimated beam with an XStrahl RS 320 cabinet irradiator (XStrahl Limited, Camberly, Surrey, Great Britain). ABT-263 was given at a dose of 75 mg/kg via intraperitoneal injection in a total volume of 50 μL every second day starting at day of irradiation until day 10. 1 h before euthanization, 60 mg/kg pimonidazole hydrochloride (Hydroxyprobe, Burlington, MA, USA) was injected intraperitoneal for immunohistochemical analysis of hypoxic regions.

### Immunohistochemistry

Paraffin-embedded tissue sections were hydrated using a descending alcohol series, incubated for 10–20 min in target retrieval solution (DAKO, Glostrup, Denmark) and incubated with blocking solution (2% normal goat serum/PBS). After permeabilization, sections were incubated overnight at 4 °C with mouse-anti Mcl-1 (1/200; Santa Cruz, Dallas, TX, USA), mouse-anti Bcl-x (1/50, BD Bioscience, Heidelberg, Germany), or mouse-anti- Hypoxyprobe™ Mab-1 antibody for detection of pimonidazole (Hydroxyprobe, Burlington, MA, USA). Antigens were detected with a horseradish peroxidase-conjugated secondary antibody (1/250) and 3,3′-diaminobenzidine staining (DAKO) as previously described [48, 49]. Nuclei were counterstained using hematoxylin.

### Statistical analysis

Numerical data show means of at least three independent experiments ± standard deviation (SD), unless stated otherwise. The results were subjected to statistical analysis using GraphPad Prism 6 software (GraphPad Software, California, USA). Statistical significance was calculated by *t*-test or two-way ANOVA followed by Bonferroni post hoc test. *P*-value < 0.05 was considered statistically significant.

## Supplementary information

Supplementary Figure 1

## Data Availability

The datasets used and/or analyzed during the current study are available from the corresponding author on reasonable request.
