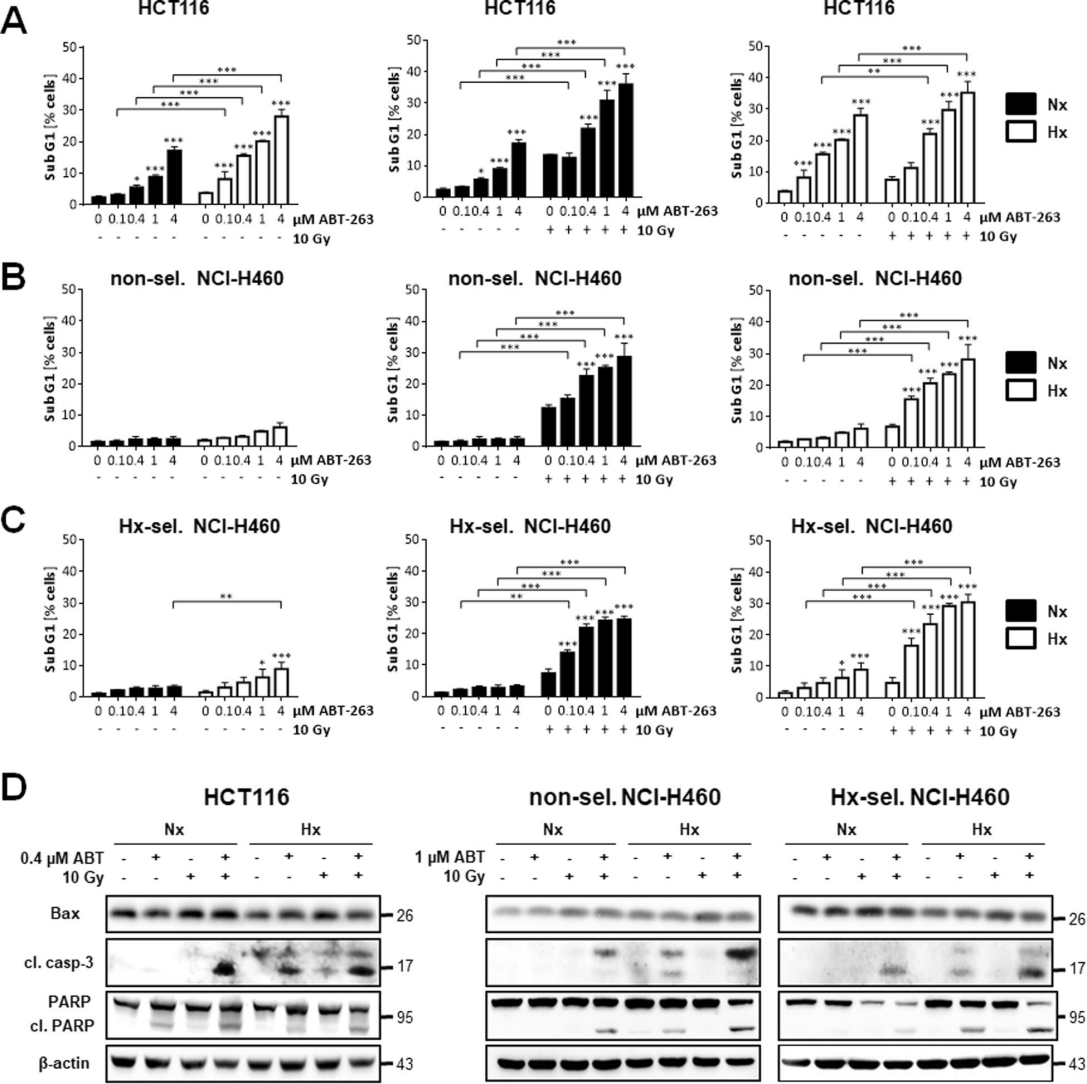# Correction to: Bcl-2/Bcl-xL inhibitor ABT-263 overcomes hypoxia-driven radioresistence and improves radiotherapy

**DOI:** 10.1038/s41419-022-04743-7

**Published:** 2022-04-19

**Authors:** Violetta Ritter, Franziska Krautter, Diana Klein, Verena Jendrossek, Justine Rudner

**Affiliations:** Institute for Cell Biology (Cancer Research), University Hospital Essen, University of Duisburg-Essen, Essen, Germany

**Keywords:** Cancer microenvironment, Radiotherapy, Cell biology

Correction to: *Cell Death and Disease* 10.1038/s41419-021-03971-7, published online 13 July 2021

The original version of this article, unfortunately, contained a mistake in Figure 2. The correct figure is given below. The original article has been corrected.