# Supplementary material for: Bcl-2/Bcl-xL inhibitor ABT-263 overcomes hypoxia-driven radioresistence and improves radiotherapy
Source: Cell Death Dis. 2021 Jul 13;12(7):694. doi: 10.1038/s41419-021-03971-7 (PMC8277842; doi:10.1038/s41419-021-03971-7)
Supplement: Supplementary file 1 — Supplementary Figure 1 [file 41419_2021_3971_MOESM1_ESM.pptx]

## Slide 1
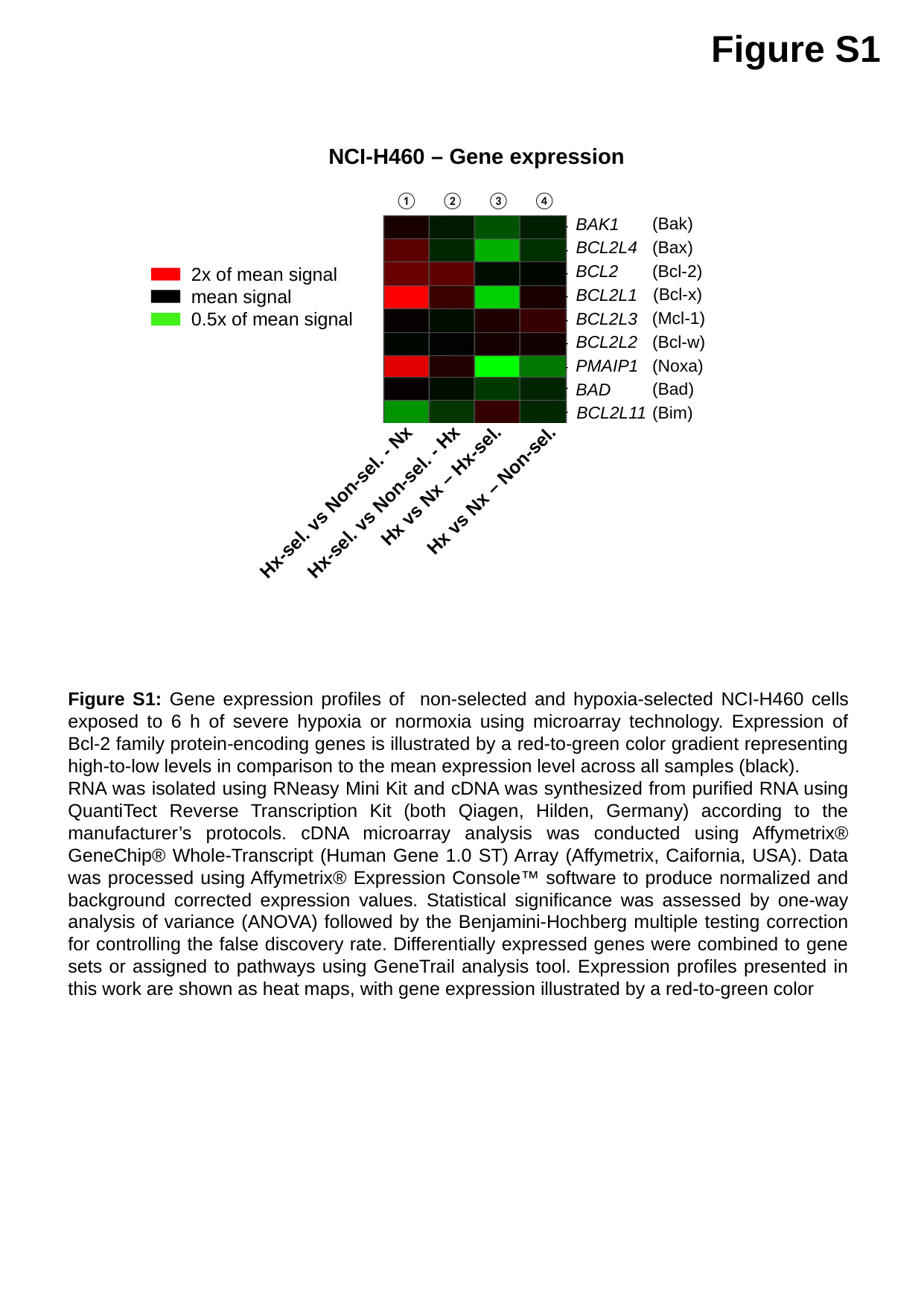

Figure S1
NCI-H460 – Gene expression
① ② ③ ④
(Bak)
BAK1
(Bax)
BCL2L4
(Bcl-2)
BCL2
2x of mean signal
mean signal
0.5x of mean signal
(Bcl-x)
BCL2L1
(Mcl-1)
BCL2L3
(Bcl-w)
BCL2L2
(Noxa)
PMAIP1
(Bad)
BAD
(Bim)
BCL2L11
Hx vs Nx – Hx-sel.
Hx vs Nx – Non-sel.
Hx-sel. vs Non-sel. - Nx
Hx-sel. vs Non-sel. - Hx
Figure S1: Gene expression profiles of non-selected and hypoxia-selected NCI-H460 cells exposed to 6 h of severe hypoxia or normoxia using microarray technology. Expression of Bcl‑2 family protein‑encoding genes is illustrated by a red‑to‑green color gradient representing high‑to‑low levels in comparison to the mean expression level across all samples (black).
RNA was isolated using RNeasy Mini Kit and cDNA was synthesized from purified RNA using QuantiTect Reverse Transcription Kit (both Qiagen, Hilden, Germany) according to the manufacturer’s protocols. cDNA microarray analysis was conducted using Affymetrix® GeneChip® Whole-Transcript (Human Gene 1.0 ST) Array (Affymetrix, Caifornia, USA). Data was processed using Affymetrix® Expression Console™ software to produce normalized and background corrected expression values. Statistical significance was assessed by one-way analysis of variance (ANOVA) followed by the Benjamini-Hochberg multiple testing correction for controlling the false discovery rate. Differentially expressed genes were combined to gene sets or assigned to pathways using GeneTrail analysis tool. Expression profiles presented in this work are shown as heat maps, with gene expression illustrated by a red-to-green color
